# Lead Toxicity: A Case Report of a Rare and Potentially Underdiagnosed Condition in Nepal

**DOI:** 10.1002/ccr3.70872

**Published:** 2025-09-05

**Authors:** Ryan McHenry, Sanjaya Karki, Pradeep Maharjan, Mirab Singh, Shruti Thapa, Rajan Basnet, Yuvraj Basnet, Neeraj Joshi

**Affiliations:** ^1^ ScotSTAR, Scottish Ambulance Service, Hangar B Paisley UK; ^2^ Nepal Mediciti Hospital Lalitpur Nepal; ^3^ Tribhuvan University Kirtipur Nepal; ^4^ Manipal Pokhara College of Medical Sciences Pokhara Nepal

**Keywords:** abdominal pain, chemically‐induced disorders, chronic lead poisoning, low and middle income countries, poisoning, public health

## Abstract

Lead poisoning is rare, but easily missed, with the highest burden of disease occurring in low‐to‐middle income countries. However, when diagnosed, appropriate treatment can prevent long‐term morbidity. A case is presented, reinforcing the importance of thorough history‐taking and international guidelines in successful treatment.

## 1 Introduction

Lead toxicity is a rare, but easily missed, diagnosis associated with widespread clinical manifestations, including neurological, gastrointestinal, hematological, and renal sequelae. Lead exposure can occur from a variety of domestic, occupational, and traumatic sources, with the highest risk occurring in low‐to‐middle income countries such as Nepal [1, 2, 3]. Lead toxicity results in widespread effects through the hematopoietic, renal, reproductive, and central nervous system, with both acute and chronic sequelae [1, 4]. The mechanism of lead toxicity is complex, mainly occurring through increased oxidative stress, with management strategies focused on the removal of relevant exposures and therapies to reduce blood lead concentrations [1, 4]. Symptoms of lead toxicity may include anorexia with weight loss, abdominal pain, behavioral changes, headache, and complications in pregnancy such as reduced fetal growth [1]. As a rare condition with multi‐system involvement, the diagnosis of lead toxicity requires thorough history‐taking, a high index of suspicion, and appropriate investigations. Here, we present a case of a patient with multiple potential sources of lead exposure, presenting with abdominal pain, highlighting the non‐specific nature of presenting symptoms, and treatment options available.

## 2 Case History and Examination

A 27‐year‐old Nepali female presented to an Emergency Department in Kathmandu, Nepal, with a one‐month history of progressive, generalized abdominal pain, with a focus in the left lower quadrant. The pain was characterized as “burning,” with a colicky pattern. Associated symptoms included reduced appetite, nausea, and nonbloody, nonbilious vomiting. She also reported intermittent constipation, which was temporarily relieved with enema administration, and experienced easy fatigability during daily activities.

There was no history of headache, altered sensorium, altered behavior, insomnia, muscle cramps, hair loss, fever, weakness of the extremities, or bloody stool. Prior to presentation at the Emergency Department, investigations at another center had suggested iron deficiency anemia and ultrasound had demonstrated a right ovarian cyst. Despite treatment with analgesia, oral antibiotics, and iron supplements, her symptoms persisted.

The patient had no significant past medical history, a normal menstrual cycle, and was a non‐smoker, though with significant passive exposure to tobacco smoke in the domestic environment. The patient reported significant family medical history including chronic abdominal pain in her mother, father, and brother. Occupational history included work in her family's business, a cosmetic store in the Kathmandu Valley, a poorly ventilated space that has undergone frequent renovations, potentially including the use of lead‐based paints. The patient described no potential for retained foreign body, including no previous surgical history, nor penetrating trauma.

On examination, she was conscious, cooperative, and orientated. Pallor was noted, but there was no icterus, cyanosis, clubbing, lymphadenopathy, or oedema. Respiratory, cardiovascular, and central nervous system examinations were unremarkable. Abdominal examination revealed minimal tenderness at the left iliac fossa, but without rigidity or guarding.

## 3 Differential Diagnosis, Investigations, and Treatment

Initial investigations revealed hemoglobin levels of 8.3 g/dL with blood film demonstrating normocytic normochromic red blood cells and anisocytosis but no basophilic stippling. Liver enzymes were moderately elevated (ALT 153 U/L). Urinalysis showed pus cells, and culture isolated *Enterococcus* species. Upper gastrointestinal endoscopy indicated antral gastritis, and abdominal ultrasonography identified a small left corpus luteal cyst. Computed tomography (CT) of the abdomen was normal. Blood lead levels, requested due to the lack of definitive diagnosis from these investigations and clinical suspicion of lead exposure, were above the upper limit of detection in the hospital laboratory of 85 μg/dL, confirming lead poisoning. No other heavy metal assay was available.

The patient was treated symptomatically for pain and nausea with a single dose of intravenous tramadol 50 mg and ondansetron 4 mg. On discharge, the patient was provided with oral tramadol 50 mg as required for pain, with cefixime 200 mg twice daily for 2 weeks for urinary tract infection, and lactulose 3.35 g/5 mL, 30 mL as required for constipation. In line with international guidelines [1], the patient met the threshold for treatment with a chelation agent and was discharged with penicillamine 250 mg (5.5 mg/kg) twice daily for 2 days then three times daily for 1 month.

Appearances on ultrasound were felt to be unrelated to lead toxicity and required no treatment. Gastritis, identified on upper gastrointestinal endoscopy, was treated with oral pantoprazole 40 mg once daily for 4 weeks.

## 4 Conclusion and Follow‐Up

At the 1‐month follow‐up, blood lead levels were 31.3 μg/dL, and the patient reported complete resolution of symptoms, with no ongoing abdominal pain or nausea, and a restoration of normal appetite. Given the regional context, with limitations on access to care, no further follow‐up was indicated.

## 5 Discussion

This case report demonstrates the non‐specific nature of presenting complaints often associated with lead toxicity, and the value of thorough history‐taking to elicit occupational exposure to potential poisonous agents, in line with other reports from the literature [3]. In this case, multiple potential sources of lead exposure were identified, in particular from occupational handling of cosmetics, and exposure to lead‐based paints, both significant contributors to lead exposure worldwide and locally in Nepal [1, 2, 5, 6]. Reflecting the uncertainty regarding the definitive source that may often be encountered in clinical practice, the patient was advised to modify each of the potential exposures; eliminating contact with lead‐based products from their cosmetics business and to seek alternative sources of paint during renovations. The clinical presentation of lead poisoning is highly variable, and there is wide variation in the dose required before symptoms develop [7]. Even very low blood lead levels of < 5 μg/dL are associated with decreases in cognitive performance and academic achievement in children [1, 8].

While no comprehensive national data is available, lead exposure has been identified as an important public health concern in Nepal, with evidence of elevated blood lead levels in 64.4% of children attending outpatient departments in Kathmandu Valley [2], and high lead levels have been described in cosmetics available in the region [9]. There have been case reports of lead toxicity arising from cosmetics use for over a century [10], and other case reports from the region have described abdominal pain as a presenting complaint for the condition [11]. The most important sources of lead exposure are highly variable depending on country and context [1], though data from the United States of America demonstrates that manufacturing industries are the primary source of exposure, with 70% of those exposures relating to the manufacture of storage batteries [12]. Data from the South Asia region identifies agrochemicals and foodstuffs as important occupational and domestic sources of lead exposure [13].

While international guidelines consider multiple chelating agents, they note that penicillamine is the most likely to be available in the low‐to‐middle income countries with the highest burden of lead toxicity [1]. Notable limitations of penicillamine include sparse evidence in severe toxicity, and risk of teratogenicity in the first trimester, when it should be avoided. No chelating agents have been determined as safe in early pregnancy, and guidelines suggest the benefits and risks of treatment must be carefully balanced. The optimal duration of treatment with penicillamine is not indicated in international guidelines, but low‐certainty evidence suggests 5 or 10 day courses of four‐times‐daily penicillamine at 250 mg doses sufficiently reduce blood lead concentrations and symptoms [14].

Ultimately, reductions in the burden of lead toxicity will only occur with concerted efforts in public health; with the introduction and enforcement of restrictions in the ongoing use of lead and removal of lead‐based products already present in homes and workplaces. International policy documents recognize the challenges that may be associated with efforts to reduce the harms from lead exposure, particularly in communities that have little agency to improve their environmental conditions or are dependent on lead‐based products for their economic livelihoods [15]. However, it is important to note that low‐to‐middle‐income countries have made efforts to mandate reductions in lead exposure, such as recent laws to restrict the presence of heavy metals, such as lead, from foods in Nepal [16].

This case demonstrates the importance of considering lead poisoning in patients with non‐specific symptoms, particularly in regions with known environmental risks. Comprehensive history taking, including environmental and occupational exposures, is crucial in reaching the diagnosis. Effective management involves both medical intervention and addressing sources of exposure. Public health measures remain essential in preventing lead poisoning and protecting vulnerable populations, particularly children.

## Author Contributions

**Ryan McHenry:** methodology, writing – original draft, writing – review and editing. **Mirab Singh:** resources. **Pradeep Maharjan:** project administration. **Sanjaya Karki:** conceptualization. **Shruti Thapa:** resources. **Rajan Basnet:** resources. **Yuvraj Basnet:** writing – review and editing. **Neeraj Joshi:** supervision.

## Ethics Statement

The case report submission was supported by the Institutional Review Board of Mediciti Hospital, Kathmandu.

## Consent

The patient provided written and verbal consent, which is available with the publisher.

## Conflicts of Interest

The authors declare no conflicts of interest.

## Data Availability

The case report data contains confidential patient information; anonymised data is available for review.
